# Bio-Based Solvents and Gasoline Components from Renewable 2,3-Butanediol and 1,2-Propanediol: Synthesis and Characterization

**DOI:** 10.3390/molecules25071723

**Published:** 2020-04-09

**Authors:** Vadim Samoilov, Denis Ni, Arina Goncharova, Danil Zarezin, Mariia Kniazeva, Anton Ladesov, Dmitry Kosyakov, Maxim Bermeshev, Anton Maximov

**Affiliations:** 1 A.V. Topchiev Institute of Petrochemical Synthesis, Russian Academy of Sciences (TIPS RAS), 29 Leninsky Prospect, 119991 Moscow, Russiamax@ips.ac.ru (A.M.); 2 Core Facility Center “Arktika”, Northern (Arctic) Federal University, 17 Severnaya Dvina Embankment, 163002 Arkhangelsk, Russia; lokoal13@gmail.com (A.L.); d.kosyakov@narfu.ru (D.K.)

**Keywords:** renewable solvents, ketals, ethers, 2,3-butanediol, renewable fuel, propylene glycol, Kamlet-Taft

## Abstract

In this study approaches for chemical conversions of the renewable compounds 1,2-propanediol (1,2-PD) and 2,3-butanediol (2,3-BD) that yield the corresponding cyclic ketals and glycol ethers have been investigated experimentally. The characterization of the obtained products as potential green solvents and gasoline components is discussed. Cyclic ketals have been obtained by the direct reaction of the diols with lower aliphatic ketones (1,2-PD + acetone → 2,2,4-trimethyl-1,3-dioxolane (TMD) and 2,3-BD + butanone-2 → 2-ethyl-2,4,5-trimethyl-1,3-dioxolane (ETMD)), for which the Δ*H*^0^_r_, Δ*S*^0^_r_ and Δ*G*^0^_r_ values have been estimated experimentally. The monoethers of diols could be obtained through either hydrogenolysis of the pure ketals or from the ketone and the diol via reductive alkylation. In the both reactions, the cyclic ketals (TMD and ETMD) have been hydrogenated in nearly quantitative yields to the corresponding isopropoxypropanols (IPP) and 3-sec-butoxy-2-butanol (SBB) under mild conditions (*T* = 120–140 °C, *p*(H_2_) = 40 bar) with high selectivity (>93%). Four products (TMD, ETMD, IPP and SBB) have been characterized as far as their physical properties are concerned (density, melting/boiling points, viscosity, calorific value, evaporation rate, Antoine equation coefficients), as well as their solvent ones (Kamlet-Taft solvatochromic parameters, miscibility, and polymer solubilization). In the investigation of gasoline blending properties, TMD, ETMD, IPP and SBB have shown remarkable antiknock performance with blending antiknock indices of 95.2, 92.7, 99.2 and 99.7 points, respectively.

## 1. Introduction

The use of fossil fuel feedstocks for the production of energy carriers and chemicals has been developed a lot during the XXth century, and can now be considered as one of the main sources of current ecological problems. Among the latter there are environmental pollution (particularly, air, water and soil contamination) and climate change, which influence each other. One of the possible solutions to the aforementioned problems could be the extensive development of the biomass processing industry that provides the chance to replace fossil-based fuels and chemicals with bio-based renewable analogues.

Renewable glycols such as 1,2-propanediol (1,2-PD) and 2,3-butanediol (2,3-BD) are of great interest as potential starting materials for chemical conversions for several reasons. First, they can be readily obtained from biomass sources by different methods: 1,2-PD can be obtained either by bioglycerol hydrogenolysis [1,2] or by microbial production starting from carbohydrates [3]. The process of carbohydrate biomass fermentation, which yields 2,3-BD, is remarkable as it provides a relatively high diol yield as well as overall productivity along with a quite low energy consumption. The low host toxicity of 2,3-BD which is conducive to a higher possible product titer in the fermentation broths should be also mentioned [4,5,6,7]. The direct microbial conversion route of CO_2_ into 2,3-BD is also worth mentioning [8]. Hence, the noted compounds are of particular interest as renewable feedstocks for the production of chemicals.

**Scheme 1:**
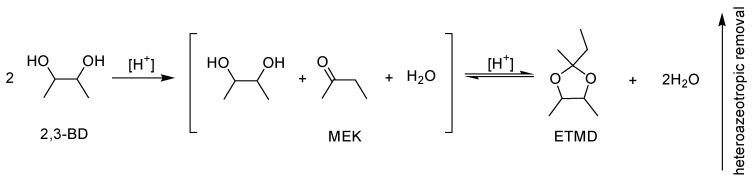
One step-conversion of 2,3-BD into a cyclic ketal.

The majority of studies on 2,3-BD conversion are dedicated to its dehydrative conversion that yields methyl ethyl ketone (MEK) and isobutyraldehyde [9,10,11,12], 3-butene-2-ol [10,13,14,15], butenes [16,17,18] and 1,3-butadiene [19,20,21]. Some 2,3-BD derivatives have been previously considered as potential motor fuel components and organic solvents. As far as this aspect is concerned, one should note the recent work by Harvey [22], which has arisen interest to the one-step 2,3-BD dehydrative conversion into a cyclic ketal by condensation with MEK generated in situ (Scheme 1), previously developed by Neish et al. in 1945 [23].

The main feature of the abovementioned approach to 2,3-BD conversion is the continuous stripping of the reaction products (water and the cyclic ketal) out of the reaction mixtures, which may provide favorable conditions for driving the reversible ketalization reaction to completion. The one-step conversion might also be partially combined with the drying of 2,3-BD, since some water content in the feed diol does not decrease the target product yield. Nearly quantitative yields of the ketal could be obtained in this process by applying a proper combination of process conditions such as temperature, pressure and acid catalyst concentration [23].

The cyclic ketal derived from 2,3-BD — 2-ethyl-2,4,5-trimethyl-1,3-dioxolane — has been characterized as a gasoline component and an organic solvent by Harvey et al. [22]. As a gasoline additive, the compound is significant for its good miscibility with fuel, high lipophilicity, and acceptable boiling point (132–142 °C depending on the isomeric composition), relatively high antiknock performance (RON/MON = 93.5/86.7) and calorific value (28.3 MJ L^−1^ versus 21.1 MJ L^−1^ for ethanol). Although the use of ETMD as a renewable solvent has been proposed, the existing data is likely insufficient for understanding its potential. In addition, in the aforementioned study by Harvey et al., some properties of the pure compound including the neat octane numbers were reported without the blending octane numbers and without any detailed data on the base gasoline properties. Some data on the diesel fuel blending properties of 2,3-BD acetals had also been reported earlier by Staples et al. [24]. Thus, a deeper investigation of both solvent and gasoline-blending properties of the 2,3-BD ketal derivatives is reasonable for exploring its real application potential.

Renewable 1,2-PD could also be transformed into a cyclic ketal via traditional acid-catalyzed ketalization with acetone [25]. It should also be noted that the formation of TMD in the vapor-phase hydrogenolysis of solketal has been recently reported [26]. Some data on 1,2-PD cyclic ketals regarding their diesel fuel blending and physical/solvent properties can be found in the literature [25,27], whereas no data on the gasoline blending properties could be found by us.

**Scheme 2:**
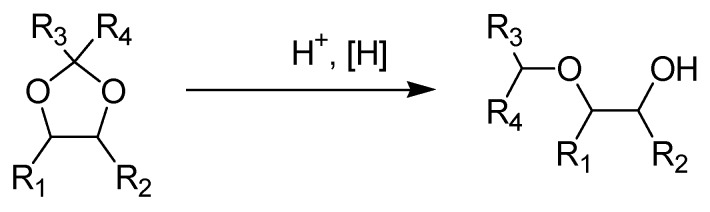
Synthesis of the glycol ethers by hydrogenolysis of the corresponding cyclic ketals.

The combination of the ketalization process with the non-destructive ketal hydrogenolysis reaction that yields the corresponding ethers (Scheme 2) offers intriguing prospects.

This reaction can be conducted with various reductive agents such as LiAlH_4_-AlCl_3_ [28,29,30,31], LiAlH_4_-BF_3_ [32], NaBH_4_ [33,34], TMDS [35,36] and hydrogen [37,38,39,40]. In the latter case a bifunctional catalyst system is required, consisting of either a heterogeneous Pd/C hydrogenation catalyst in combination with a homogeneous acid (*p*-TSA or camphorsulfonic acid) or one based on palladium supported on an acidic aluminosilicate or an acidic carbon [41,42]. When employing hydrogen as a reducing agent (what can be considered the most feasible route) nearly quantitative ether yields could be achieved. In this manner, the renewable diols could be converted into the corresponding cyclic ketals and then they could be subjected to selective hydrogenolysis that yields renewable glycol ethers. The compounds of the latter group are the well-known organic solvents and hydrotropes with multiple applications [43,44]. A variation of the approach of interest is the direct reductive alkylation reaction between a diol and a carbonyl compound. In this case, the synthesis of the glycol ether can be conducted in a one-step process under mild conditions (e.g., under moderate hydrogen pressure of 1–4 MPa and 100–140 °C) [38,40]. A number of recent papers were dedicated to the conversion of renewable diols, namely, 1,2-propanediol and glycerol, into ethers and ketals, as well as to the characterization of the mentioned derivatives as potential renewable fuel components [45] and solvents [27,43,46]. Thus, the development of approaches for the synthesis of bio-based glycol ethers might be of interest for the renewable petrochemical substituents production.

The purposes of the study reported herein are: (a) to investigate the regulations of the synthesis of cyclic ketals from diols (1,2-PD and 2,3-BD) and ketones (acetone and MEK); (b) to describe and to perform some experimental evaluations of approaches to glycol ethers synthesis either by cyclic ketal hydrogenolysis or via reductive alkylation; (c) to characterize the cyclic ketal and glycol ether derivatives regarding their potential applicability as organic solvents and gasoline components; and (d) to perform a primary sustainability evaluation of the proposed routes towards bio-based solvents.

## 2. Results and Discussion

### 2.1. The Ketalization of 1,2-PD and 2,3-BD with Acetone and MEK

The ketalization between diols and acetone (or MEK) should comply with the general rules known for the reactions of this type. As is known, the ketalization process is slightly exothermic and results in a decrease in entropy [47]. Thus, to obtain the maximum ketal yields lower temperatures are preferred. At the same time, the thermodynamic stability of the cyclic ketal products depends on the molecular structure of the precursor diols and carbonyl compounds, what is supported by the different equilibrium yields obtained either within the reactions of the diol with the different carbonyl compounds [48,49,50] or in transacetalization (transketalization) reactions [51]. In order to determine the thermodynamic equilibrium for the reactions of interest (yielding the corresponding cyclic acetals from 1,2-PD and 2,3-BD) and to evaluate the relation between the reactants molecular structure and the reaction thermodynamic, experimental measurements of the equilibrium constant temperature dependence were conducted.

**Figure 1:**
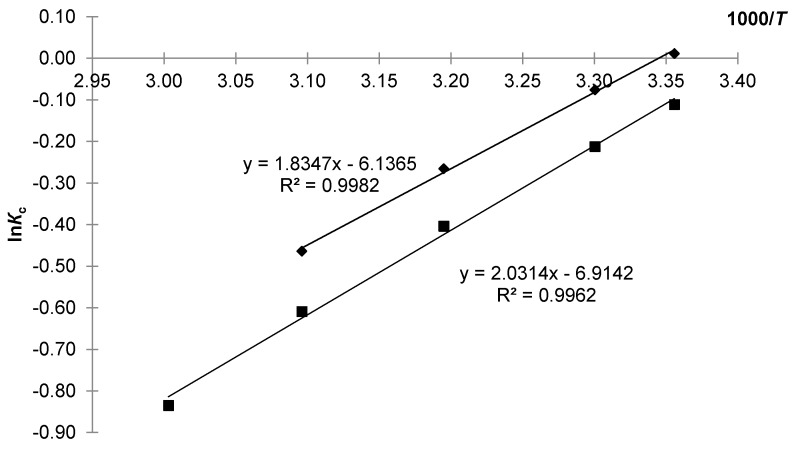
The influence of the reaction temperature on the equilibrium constant in the reactions of 2,3-BD with MEK (■) and in the reactions of 1,2-PD with acetone (♦).

The determination of equilibrium compositions was carried out under the conditions employed earlier for the ketalization between acetone and glycerol [52,53]. During the analysis of the reaction mixtures, no byproducts were observed. The experimental results obtained (Table 1) show the dependence between the equilibrium yield and the temperature typical for the homologous ketalization reactions. The data was plotted in the Arrhenius coordinates (Figure 1) and fitted with a linear function with good precision.

Using the Van ’t Hoff equation in the form:

(1) $$lnK_{c}=\frac{\Delta S^{0}}{R}-\frac{\Delta H^{0}}{R}\frac{1}{T}$$

and the equation which expresses the relation between the changes Gibbs free energy, the enthalpy and the entropy upon the reaction:

(2) $$\Delta G^{0}=\Delta H^{0}-T\Delta S^{0}$$

the corresponding values of Δ*G*^0^, Δ*H*^0^ and Δ*S*^0^ for the reaction were calculated (Table 2).

A comparison between the values obtained in this work with the previously reported ones permits us to conclude that in the present case the differences in the molecular structure do not affect the thermodynamic stability of the cyclic ketals formed. The only result which shows a significant difference seems to be the result of Nanda et al. [52] for the reaction of glycerol with acetone, probably due to the use of ethyl alcohol as solvent, while other results were obtained in solventless reactions. The values of Δ*H*^0^_r_ and Δ*S*^0^_r_ obtained for the reaction between 1,2-PD and acetone by Anteunis and Rommelaere are likely to be underestimated, what might be connected with the known precision limits of the NMR measurements. At the same time, the data from the Table 2 proves the postulate of Anteunis and Rommelaere about the isoequilibrium relationship for the homological reactions of the cyclic ketal formation. For those typical values of Δ*H*^0^_r_, Δ*S*^0^_r_ are about −17.2 ± 2.6 kJ mol^−1^ and 57.2 ± 7.3 J mol^−1^ K^−1^, respectively.

The equilibrium yield values for the reactions of 2,3-BD and 1,2-PD ketalization are close to the values measured for glycerol ketalization under the similar conditions [52,53]. Pure TMD and ETMD samples were obtained via the direct ketalization under the same ketone molar excess (6:1) conditions. The isolated yields of TMD and ETMD (83.7 and 81.6%) turned out to be close to the equilibrium yields determined by GC (89.7 and 84.9%), hence the isolated yields for TMD and ETMD amounted to 0.933 and 0.961 of the corresponding equilibrium yields. Thus, the direct synthesis approach offers excellent yields along with a relatively simple set-up, and thus might be further potentially developed with a view to an industrial process. The thermodynamic data obtained here could be of interest for the necessary reaction engineering purposes.

### 2.2. The Glycol Ethers Synthesis Via the Hydrogenolysis of the Corresponding Cyclic Ketals

The properties of the catalysts used for 1,2-PD and 2,3-BD monoether synthesis are given in Table 3. The performance of Pd/Al-HMS materials in the cyclic glycerol ketal hydrogenolysis reaction and the characterization details have been previously reported [39], so in this paper only the brief description is given. The values of the specific surface areas for the catalysts indicate well-developed pore structures (between 680 m^2^ g^−1^ and 850 m^2^ g^−1^) and a narrow pore size distribution with mean pore sizes of about 3–5 nm. The acidity of the mesoporous aluminosilicas demonstrates a non-linear growth with the increase in the Al content. The acidity strength distribution pattern for Al-HMS aluminosilicas determined previously has shown that strong acid sites are practically absent, what might provide the high selectivity in the reaction of the cyclic ketals hydrogenolysis [39].

The palladium particles are well dispersed, with mean sizes between 3 nm and 5 nm, what allows us to reach the conclusion that the metal particles size seems to be limited by the pore size and that the metal particles are predominately located inside the pores of the Al-HMS. The metal particles dispersion degrees in the catalysts with the supports of the different Si/Al ratio are practically identical.

**Scheme 3:**
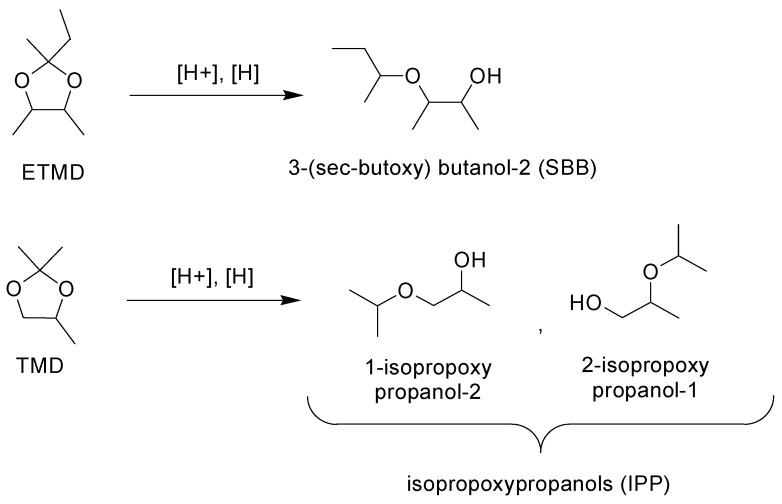
The hydrogenolysis of the cyclic ketals (the reaction scheme).

The catalysts were tested in the cyclic ketal (both TMD and ETMD) hydrogenolysis reaction yielding the corresponding glycol ethers (Scheme 3). The reaction is known for its high selectivity: the yields are usually nearly quantitative for the reduction with hydrogen [38,41], LiAlH_4_ [55] or 1,1,3,3-tetramethyldisiloxane [36]. The results of the catalytic tests (Table 4) demonstrate that in the present case the selectivity values were also very high, normally reaching 97–98 mol %. The corresponding glycol ether (IPP for TMD, SBB for ETMD) was actually the main and only reaction product with the remaining 2–3% of selectivity accounted for by the hydrolysis products (the ketone and the diol), originated from the trace water.

The hydrogenolysis of the glycerol-acetone cyclic ketal (solketal) over these catalysts has been studied earlier. It has been revealed by the authors that the Si/Al ratio of the support influences the overall catalytic activity [39]. The observed differences were attributed to the different activity of the supports in the acid-catalyzed reactions, e.g., ketalization, as more hydrophobic supports with greater Si/Al ratio showed to be more active. The strong adsorption of polar species (for example, water, polar organic compounds), which are typical for the relatively hydrophilic materials, might be the reason for their limited catalytic activity [50,52]. Thus, in the case of solketal catalytic hydrogenolysis, more hydrophilic material is likely to adsorb the primary hydrogenolysis product that is the diol.

In the present case, the authors have not observed any significant differences between the palladium catalysts supported over the Al-HMS materials with the Si/Al ratios ranging from 10 to 30 (Table 4). The Al-HMS(10) catalyst was slightly less active at the lower temperature, while the activities of all the catalysts under *T* = 160 °C were practically of the same value. This lets one suppose that in the present case there is no significant correlation between the support hydrophobicity and the catalytic activity, since the difference in the polarity of the reactant and the product (the ketal and the corresponding ether) is likely to be relatively low. As shown below, the physical properties in the “diol ketal–glycol monoether” pairs (for example, the water miscibility, boiling points and vapor pressures) are much closer compared to the “solketal–glycerol monoisopropyl ether” pair.

By conducting the reaction for more time, a nearly quantitative yield of the ether might be obtained. Under the optimized conditions (Table 4, entry 5) both the TMD and ETMD were converted into the corresponding ethers with yields over 90 mol % (91.4 and 92.1 mol %, respectively), thus indicating at the feasibility of the approach for the catalytic synthesis of the renewable glycol ethers.

For the synthesis of ethers via the catalytic hydrogenolysis of the corresponding acetals and ketals high regioselectivity values are typical [39,40]. While the hydrogenolysis of ETMD yields only one structural isomer, the hydrogenolysis of TMD could yield two isomeric 1,2-PD monoethers: 2-isopropoxy-1-propanol and 1-isopropoxy-2-propanol. According to our results, the regioselectivity for 1-isopropoxy-2-propanol reached 87–91%. This value is lower than the previously observed one in solketal hydrogenolysis and it is closer to the regioselectivity of the solketal *O*-isopropyl ether reduction [39]. Close regioselectivity values have been also reported for the direct reductive alkylation of glycerol with aliphatic aldehydes [41]. In the preparative syntheses carried out in the given work upon the reduction of TMD in the LiAlH_4_-AlCl_3_ system the regioselectivity for the 1-isopropoxy-2-propanol was about 82%, being here typical for this approach [29].

**Scheme 4:**
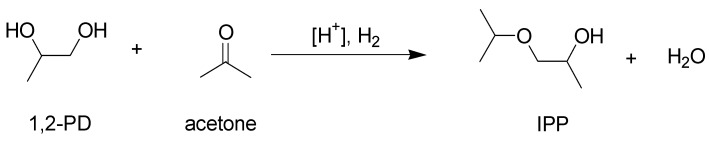
The reductive alkylation of acetone with 1,2-PD over a bifunctional catalyst.

Since ETMD might be obtained from 2,3-BD with the excellent yield in the one-step process, this compound seems to be the more preferable one as the starting material for the synthesis of the corresponding glycol ether by the pure ketal hydrogenolysis comparing with 2,3-BD. The renewable 1,2-PD may be obtained by the catalytic hydrogenolysis of bioglycerol. 1,2-PD cost is comparable to the price of acetone, so the feasibility of the one-step conversion of 1,2-PD to TMD in the manner that is similar to 2,3-BD conversion to ETMD seems to be controversial. Thus, the conversion of 1,2-PD into the corresponding glycol ether by reductive alkylation with acetone (the latter can originate from sugars by the ABE fermentation) seems to be of value (Scheme 4).

The reductive alkylation reaction is generally believed to run through the formation of the intermediate ketal followed by its catalytic hydrogenation [38]. Therefore, the influence of the reaction temperature should be crucial: its increase enhances the hydrogenolysis rate, while decreasing the equilibrium concentration of the intermediate ketal described above in the ketalization thermodynamic study. The experimental results (Table 5) have shown that there is a temperature optimum for the reductive alkylation reaction. For example, upon the reductive alkylation of 1,2-PD with acetone under *T* = 120, 140, 160 °C (Table 5, entries 7–9), the yields of IPP amounted to 15.2, 58.7 and 7.9%, respectively. Hence, at the lower temperature the ether yield decreased along with the hydrogenation rate. At 160 °C the obtained ether yield was connected with the unfavorable influence of the temperature on the equilibrium between diol and the corresponding ketal, which is the reaction intermediate. The decrease of the diol:ketone ratio from 40:1 to 20:1 (mol/mol) (Table 5, the entries 7 and 12) also led to an ether yield decrease (from 15.2 to 6.8%), thus supporting the determinant influence of the ketal equilibrium concentration on the target product yield. Regarding the reaction with the greater reaction time, the glycol ether yields are likely to be as high as 92.6 and 93.3 mol % for IPP and SBB, respectively (Table 5, entries 5 and 11). The regioselectivity between the IPP structure isomers was close to the values obtained in the direct TMD hydrogenolysis (about 90–91% to 1-isopropoxy-2-propanol).

The reusability of the bifunctional catalyst was checked in both the pure ETMD hydrogenolysis and 2,3-BD + MEK reductive alkylation reactions (Table 6). After the first ketal hydrogenolysis cycle a slight increase in the catalyst activity was observed (the SBB yield increased from 35.6 to 36.9 mol %), probably, due to the pre-reduction of the catalyst. For the subsequent cycles, a slight decrease in activity was observed: in the fifth cycle, the target product yield amounted to 92.5% of the initial value. In the case of the 2,3-BD reductive etherification with MEK, after the first cycle the target product yield decreased sharply from 62.6 to 54.7 mol %. In the next cycles, the activity change was lower, despite the fact that the SBB yield gradually decreased to 52.1 mol % (83.8% of the initial value). This pattern of the activity change observed in the latter reaction might be attributed to the adsorption of water on the catalyst active sites: the fresh catalyst sample showed the higher activity as it was dry, while the spent catalyst contacted with the water that was released in the catalytic reaction. It should be taken into the account that the presence of water might influence the reductive etherification by the affecting on the ketalization equilibrium [39] or the palladium reduction process [42].

Based on the results obtained, these approaches, namely the hydrogenolysis of the pure ketal obtained in the separate synthesis from the diol and the ketone, and the one-step reductive ketalization alkylation, both turned out to be feasible for the synthesis of the renewable glycol ethers. The use of bifunctional Pd/Al-HMS catalysts permits the reactions to be carried out under relatively mild conditions (T = 120–140 °C) with excellent selectivities (97–98 mol % and 94–95 mol % for the ketal hydrogenolysis and the reductive ketalization alkylation) and in nearly quantitative yields. The recovery of the target product from the reaction mixtures is likely to be easily conducted by simple distillation.

### 2.3. The Characterization of the Products

The search of the new bio-based organic solvents for the substitution of the petrochemical-derived ones is an important problem of sustainable chemistry. Investigations of solvent properties of compounds derived from propylene glycol, glycerol, levoglucosane and isosorbide are to be mentioned herein as the examples [27,43,44,46,56,57]. In the given study, the efforts have been taken to describe the properties of the synthesized compounds, namely, of two cyclic ketals—TMD and ETMD and of two corresponding glycol ethers—IPP and SBB. The estimate of the properties which might be relevant for the organic solvents was performed with the respect to the criteria for the green solvents recognizing reported earlier by Jessop [58].

All the compounds tested appear to be the low-viscosity liquids with low melting points (<−60 °C) and the pleasant fruity smell. The value of the TMD viscosity is in good accordance with the data reported earlier by Kapkowski et al. [25]; it might be compared to the kinematic viscosity of *n*-butyl acetate (0.78 mm^2^ s^−1^), while the viscosity of ETMD is of the same order as that of diethyl ether (0.31 mm^2^ s^−1^). The viscosities of IPP and SBB are close to the value of 2-butoxyethanol (3.64 mm^2^ s^−1^). The boiling points values (Table 7) being less than 250 °C under atmospheric pressure place these compounds in the VOC group according to the EU classification.

**Figure 2:**
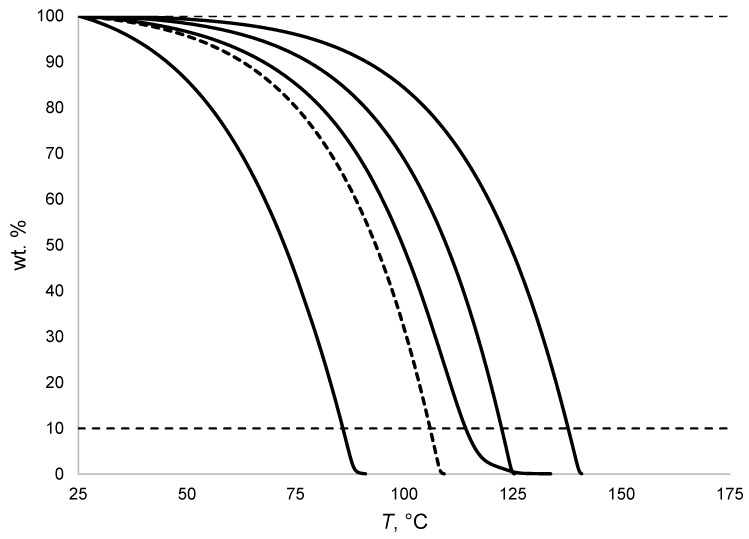
The TGA evaporation curves for IPB and SBB. From left to the right: TMD, BuOAc, ETMD, IPP, SBB.

For volatile organic compounds not only the boiling points, but also the saturated vapor pressure and the evaporation rate values are important. In the given study, the evaporation rates were estimated according to the thermogravimetric method, whose applicability was demonstrated earlier by the Aubry group [59]. The results (Figure 2, Table 8) demonstrate that TMD evaporates at the greater rate than the reference BuOAc (the evaporation rate is 1.30), while other compounds evaporate slightly slower (ETMD – 0.91, IPP – 0.84 and SBB – 0.73). The evaporation rates of the ketals might be expected to be higher than the values obtained for the corresponding ethers.

In order to characterize the volatility, the temperature dependences of saturated vapor pressure have been determined experimentally (Table S1). The coefficients of the Antoine equation have been calculated (Table 9) by means of the mathematical regression of the experimental results. It has been reported for the case of glycol and glycerol monoalkyl ethers that the TGA-derived evaporation rates correlate with the saturated vapor pressure values, but not with the boiling points [59]. In the given study the linear correlations have been observed between both the boiling point–evaporation rate (RSD = 0.984) and the saturated vapor pressure–evaporation rate (RSD = 0.977) (Figures S1 and S2). One should note that the results of the TGA evaporation rate measurements should be employed only for the primary qualitative estimate: for the isomer of IPP with the close boiling point (propylene glycol *n*-propyl ether C_3_P_1_, bp = 149 °C), a RER of 0.56 was reported [59], which is about a third lower than the value obtained by us. Moreover, the reported RER value for C_3_P_1_ showed to be about 23% lower than the one obtained for SBB, which has the highest boiling point among the compounds tested. At the same time, the experimental SVP values (at 50 °C) for IPP (1956 Pa) and its isomer, propylene glycol *n*-propyl ether 1756 Pa) [59], are in the proper relation.

All the compounds tested appeared to be completely miscible with methanol, ethanol, isopropyl alcohol, diethyl ether, toluene and dodecane. Except for IPP, the compounds were slightly miscible with water (Table 10): the miscibility with water decreased with the increase in alkyl substituents molecular weight.

The miscibility with water measured for the ketals turned out to be in all the cases lower than that one of the corresponding glycol ethers. IPP being miscible with water is likely to be considered as the component of the low-melting water-based liquids, that is why for this compound the properties of aqueous solutions have been estimated (Table 11).

One should note that if a liquid with the freezing point of −15 °C is needed, the IPP-based aqueous solution might contain less organic matter (22 vol. %) than in case of ethylene glycol and propylene glycol (both 30 vol. % for −14 °C), glycerol and isopropanol (both 40 vol. % for −15 °C). At the same time, if a lower freezing temperature is required, the only advantage of IPP over propylene glycol is a lower solution viscosity; the lower flash point of IPP compared to 1,2-PD should be considered in this case disadvantageous.

One of the major areas of organic solvents usage is the dissolution of polymers as the search for the new greener solvents for the preparation of the polymer solutions, e.g., for dyes and coatings applications is of great interest. The ketals, TMD and ETMD, turned out to be the excellent solvents for PS and polybutadiene (Table 12): 300 mg of the polymer per 1 mL of the solvent were completely dissolved to give a clear solution, and only the high viscosities of the solutions hindered a further increase in the test polymer concentration. The same polymers appeared to be just slightly soluble in the glycol ethers, which bear OH-groups in the molecular structure. The chlorinated poly(vinylchloride) (CPVC) sample, which has the high solubility in dichloroethane, might dissolve in the ketals and the glycol ethers in quite the low concentrations thus making our hopes that these compounds might partially substitute chlorinated organic solvents for the dissolution of chlorinated polymers fade.

**Figure 3:**
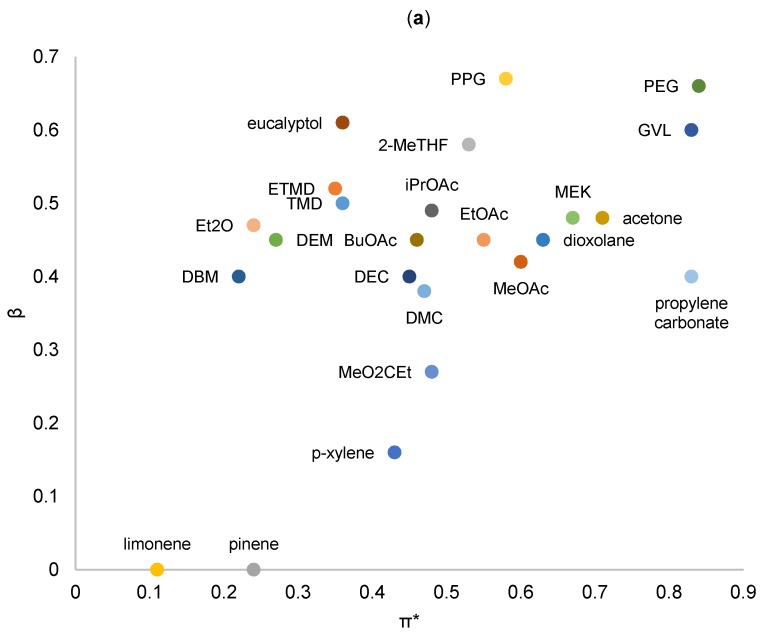

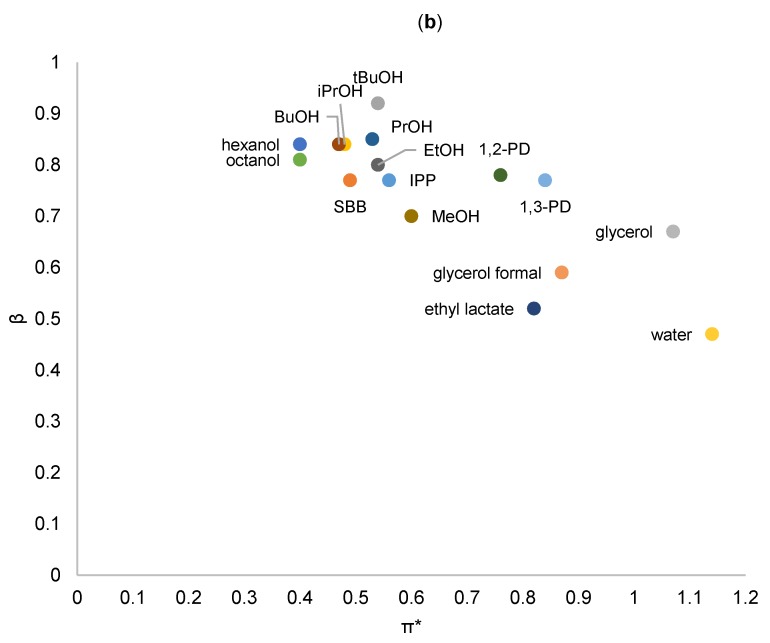
The positioning of the 1,2-PD and 2,3-BD derivatives on the β vs π* plots for aprotic (**a**) and protic (**b**) organic green solvents. The solvatochromic parameters for the rest of the compounds have been taken from the Ref. [60]. PEG—polyethylene glycol, GVL—γ-valerolactone, PPG—polypropylene glycol, DMC—dimethyl carbonate, DEC—diethyl carbonate, DEM—diethoxymethane, DBM—dibutoxymethane, 1,3-PD—1,3-propanediol, 2-MeTHF—2-methyltetrahydrofuran, MeO2CEt—methyl propionate.

The solvatochromic parameters of the renewable diol derivatives are given in Table 13. The positioning of the diol derivatives on the Kamlet-Taft β vs π* plots has been examined (Figure 3). TMD and ETMD, as far as their polarity-basicity properties are concerned, are found to be close to such the aprotic solvents as diethyl ether, *n*-butyl acetate, eucalyptol and 1,1-diethoxymethane, and thus might be related to the group of solvents with the moderate basicity and the moderate polarity (Figure 3a). The higher boiling point and the lower volatility that may propose the lower evaporation losses and the lower flammability hazards are of potential benefit over Et_2_O and DEM.

ETMD is totally comparable with BuOAc in terms of volatility; the remarkable difference between these compounds is that the former (being the cyclic ketal) is stable in basic media, while the latter undergoes rapid saponification. Thus, the ketal solvents may be employed in those cases, when an organic solvent should be used in contact with the aqueous alkali solution. One should note that it was impossible for us to measure the $E_{T}^{N}$ value for these compounds, since the Reichardt’s dye solutions gave the inadequate wavenumbers, probably due to the presence of some minor impurities, which were not obliged to detecting by neither NMR nor GC. The determination of this abnormal behavior is of the further interest for the solvatochromic characterization of the cyclic ketals.

SBB and IPP on the Kamlet-Taft plot for the protic solvents have been found among the aliphatic alcohols (Figure 3b). The boiling points of the glycol ethers are slightly lower than those of aliphatic alcohols with the same carbon atom content: the boiling points for IPP and 1-hexanol are 144 and 157 °C; 163 and 195 °C for SBB and 1-octanol, respectively. Simultaneously, in the aforementioned pairs the glycol ethers have higher miscibility with water, compared to the corresponding alcohols. While combining these two facets one can conclude that the closest alcohol analogues of IPP and SBB are 1-pentanol (bp = 138 °C, solubility in water 22 g L^−1^) and 1-hexanol (bp = 157 °C, solubility in water 6 g L^−1^), respectively. The main difference in case of IPP and pentanol is that the former is miscible with water in all the ratios. Hence, if the Ziegler process or the hydration of an oil-derived olefin is considered as the main source of fatty alcohols, IPP and SBB glycol ethers possibly have an advantage, since they are obtained from renewable resources, although 1-pentanol derived from levulinic acid might be bio-based as well.

**Figure 4:**
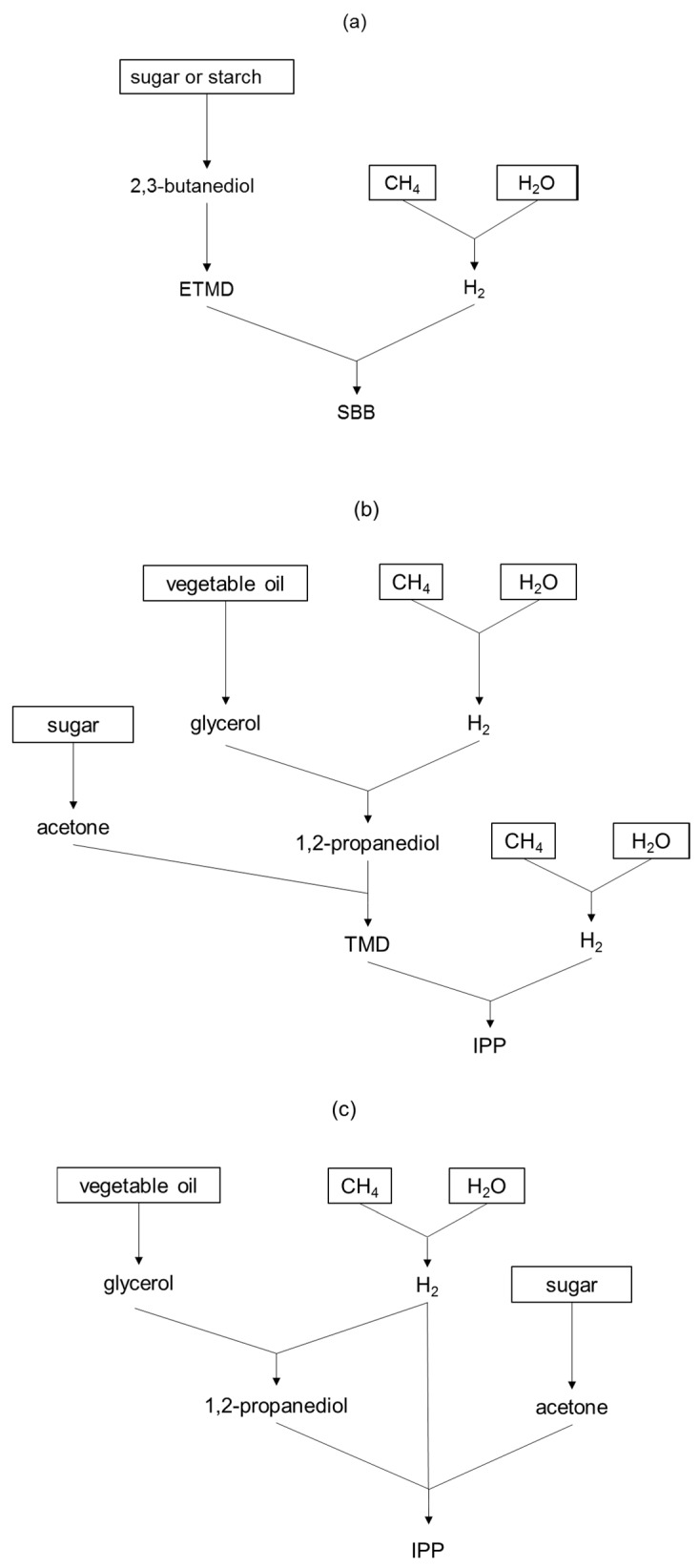
The synthesis trees for the renewable glycol derivatives: (**a**) ETMD and SBB, (**b**) IPP via the 1,2-PD ketalization and the TMD hydrogenolysis, (**c**) IPP via the reductive alkylation.

The synthesis trees for the compounds tested in the given study (Figure 4) were made up based on the following assumptions: 2,3-butanediol is obtained by the microbial fermentation of carbohydrates with the subsequent one-step conversion to ETMD, according to the protocol described by Neish [23] and Harvey [22]; SBB is obtained by the one-step hydrogenolysis of ETMD. Thus, for these compounds the number of synthetic steps is two and three, respectively (Figure 4a). The synthetic trees for 1,2-PD derivatives have been made up starting from bioglycerol-derived propylene glycol.

Although there are precedents for a one-step 1,2-PD synthesis by a retroaldol glucose conversion [61,62], currently only the glycerol hydrogenolysis process is employed on an industrial scale. Carbohydrate fermentation is supposed to be an appropriate source of the renewable acetone. The synthesis of TMD is implemented via the direct ketalization of 1,2-PD with acetone, since the feasibility of the one-step 1,2-PD to TMD conversion seems to be rather arguable in essence, representing here the dehydration of 1,2-PD to acetone. Thus, there are three synthetic steps in the TMD synthesis. For IPP obtained by the TMD hydrogenolysis it amounts to four (Figure 4b), but if the reductive alkylation route were chosen, IPP also would require only three steps to be recovered (Figure 4c).

In the final stage of our primary sustainability assessment for TMD, ETMD, IPP and SBB as organic solvents, the questions about the synthetic process formulated by Jessop [58] have been here addressed (Table 14). It is obvious that there are neither phosphorous- or nitrogen-containing wastes nor volatile heteroatomic compounds of nitrogen, sulfur and halogens in the production processes. The compounds under investigation might be considered to be fully (ETMD) or partially (TMD, IPP) renewable ones. Though providing the excellent yields and selectivity in rather mild conditions, the using of palladium catalysts for the cyclic ketal hydrogenolysis does not actually seem sustainable enough, thus employing a non-noble metal catalyst (e.g., nickel-based) for this reaction is of interest. Except the latter issue, the results of the primary sustainability estimate make it possible to suppose that the compounds investigated, particularly 2,3-butanediol-based, might be tentatively considered as potential green solvents.

As reported by Harvey et al., ETMD might have some potential as a gasoline component thanks to its appropriate volatility, relatively high calorific value and antiknock performance [22]. At the same time, there is no data on the influence of ETMD additives on the gasoline volatility properties and the antiknock performance. One can suppose that the hydrogenolysis of the cyclic ketals such as TMD and ETMD might give derivatives with increased antiknock performance, since the latter compounds bear an alcohol moiety. Alcohols are likely to be mostly efficient octane boosters when they are added to gasoline. Finally, the ketals possess relatively low stability in contact with acidic water [27,49], and despite the fact there is no data on the possible degradation of ketal-containing gasoline blends, this issue should not be disregarded. One should note here that the ethers which derive from ketal hydrogenolysis might have far higher hydrolytic stability than their precursors. Therefore, for characterizing the synthesized compounds in terms of the gasoline-blending properties the corresponding tests have been performed (Table 15). The extra purpose here was to estimate the relationships between the molecular structures of these oxygenates and their octane-enhancing efficiency.

As the tested oxygenates’ boiling points are inside the gasoline boiling range (35–193 °C), their volatilities can be considered acceptable. The main concern here is that the “excessively heavy” oxygenates can decrease the overall gasoline volatility, what could be particularly relevant for high-boiling solketal [53], γ-valerolactone [63], methyl pentanoate and alkyl levulinates [64]. The results of the fractional composition, namely, the 5 and 10 vol. % recovery temperatures allow to make the “indirect” assertion on the gasoline cold start properties. As the differences in the *T*_5%_ and *T*_10%_ between the neat and additive-containing gasolines are within the method precision, one can conclude that none of the compounds tested affect the gasoline volatility.

The blending octane numbers and the calculated AKI for ETMD (Table 15) appeared to be slightly higher than the intrinsic ONs, reported previously by Harvey et al. [22]. Other tested oxygenates showed excellent antiknock performance enhancing the knock resistance more efficiently than ETMD: the AKI values for TMD, IPP and SBB amounted to 95.2, 99.2 and 99.7 points, respectively. The impacts on the RON are not very high in comparison with, e.g., ethanol and MTBE (blending RONs about 110–120 and 115–120, respectively), whereas the calculated values of bMON are comparable. Oxygenates providing a higher effect on MON are particularly preferable, when gasoline contains significant amounts of FCC gasoline, which normally has a high octane sensitivity. As expected, TMD turned out to have a higher octane number than ETMD: for the former, there is only one atom at the tertiary carbon per molecule, while for the latter there are two, being less resistant to oxidation.

**Figure 5:**
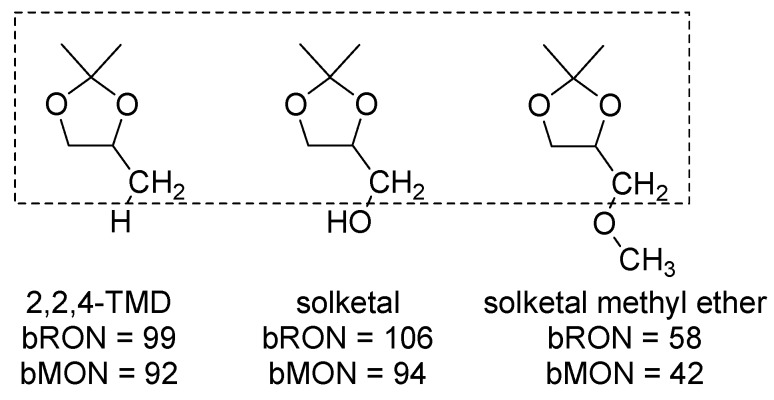
The molecular structure and the octane numbers of the 1,3-dioxolane derivatives. The octane numbers of solketal and solketal methyl ether were taken from the ref. [53].

It is of big concern that the molecular structure of TMD resembles the solketal molecule but without an OH-group. The methylation of the solketal OH-group was shown earlier to result in a dramatic ON decrease [53], and it was the OH-group (not the 2,2-dimethyl-1,3-dioxolane moiety) which was responsible for the good antiknock performance. In the present case, the octane rating of the 2,2-dimethyl-1,3-dioxolane derivative is obviously quite high. The understanding of the data on the solketal and its methylated derivative should be thus reconsidered: it seems that it is not only the positive influence of the OH-group in the solketal molecule, but the methoxy substituent is sure to affect the antiknock performance as well (Figure 5). An investigation on the relations between the molecular structure and the antiknock performance of 1,3-dioxolane homologues might be of further interest.

The mild hydrogenation of the cyclic ketals occurs with the release of the free alcohol group which might be considered as a “bearer” of the antiknock properties, as has been reported earlier [53,65]. There is little difference between the efficiency of the glycol ethers. SBB shows a slightly higher performance, although the higher impact from IPP having fewer C-H bonds at tertiary carbon atoms might be expected. However, “octane number–additive concentration” dependencies often demonstrate nonlinearity, that is why IPP and SBB might be considered to demonstrate a similar antiknock performance with the volume bMON/bRON about 101/98. The calculated molar bRON/bMON for the aforementioned glycol ethers are 101/98 and 100/97, respectively.

One should note that the volumetric calorific value of the renewable diols derivatives is quite high, compared to the alcohols with close antiknock performance. For example, 2-butanol with RON/MON = 105/93 has a density (at 20 °C) of 0.808 kg L^−1^ and a volumetric NHOC of 26.7 MJ L^−1^. The weight calorific value of SBB (32.5 MJ kg^−1^) might also be compared with that of 2-butanol and 2-methyl tetrahydrofuran (32.9 MJ kg^−1^). The SBB volumetric NHOC amounts to 28.48 MJ L^−1^ with an antiknock performance similar to that of the aforementioned alcohol [64]. Upon adding 10 vol. % of 2-butanol and SBB, the volumetric NHOC values for the blends might amount to 26.70 and 28.48 MJ L^−1^ with AKI of the both blends of 89.2–89.3 points. Thus, the loss in the overall fuel volumetric calorific value upon the adding of the octane booster is remarkably lower for SBB. What is interesting, is that both the compounds could be obtained by bio-2,3-butanediol treatment involving the dehydration with the subsequent hydrogenation. On the Van Krevelen diagram the coordinates ([O:C; H:C]) of SBB and 2-BuOH are [2.25; 0.25] and [2.50; 0.25], respectively. Thus, although alcohol has a higher hydrogen content, the efficiency of SBB as the gasoline component is higher, so one can make the conclusion that the conversion of 2,3-BD to SBB represents a method to the renewable energy recovery which might be potentially efficient and sustainable.

## 3. Materials and Methods

### 3.1. Reagents

Acetone, MEK (analytical grade, Komponent-Reaktiv, Moscow, Russia), 1,2-propanediol (>99%, Carl Roth, Karlsruhe, Germany), racemic 2,3-butanediol (Alfa Aesar, Heysham, United Kingdom, 98%), LAH (95%, Sigma-Aldrich, St. Louis, MO, USA), AlCl_3_ (Sigma-Aldrich, St. Louis, MO, USA, 99%), NaOH (pure, Komponent-Reaktiv, Moscow, Russia) diethyl ether (99.8%, Sigma-Aldrich, St. Louis, MO, USA) were used without the additional purification. The base gasoline without any oxygenates of additives was received from a Russian refinery. The properties of the base gasoline are given in Table S2 in the Supplementary Materials.

### 3.2. Preparative Syntheses

#### 3.2.1. Cyclic Ketal Syntheses

To prepare cyclic ketals (TMD and ETMD), the diol was mixed with the ketone, taken in a 6-fold molar excess, in a round-bottom flask. A dash of *p*-toluenesulfonic acid (*p*-TSA) was introduced into the flask. The mixture was stirred at room temperature for 12 h, after which the catalyst was neutralized with an excess of sodium carbonate. The reaction mixture, which consisted of the unreacted ketone and diol, water and the ketal, was subjected to rectification to obtain the pure ketal. The purity and structure of the isolated ketal was checked by GC-FID (‘Kristalluks-4000M’ gas chromatograph, Meta-Khrom, Yoshkar-Ola, Russia) equipped with a ‘Restek Rtx-Wax’ 60 m/0.53 mm/1.00 μm column, Restek, Bellefonte, PA, USA, helium as the carrier gas, pure samples used as the internal standards for the quantification, ‘Supelco SPB-1’ 30 m/0.32 mm/0.25 μm column, Supelco, Bellefonte, PA, USA, for the RI determination), GC–MS (‘Thermo Focus DSQ II’, Thermo Fischer Scientific, Waltham, MA, USA, ‘Varian VF-5ms’ 30 m/0.25 mm/0.25 μm column, Varian Inc., Palo Alto, CA, USA, helium as the carrier gas, the EI ionization), the chemical analysis (‘Thermo Flash 2000’, Thermo Fischer Scientific, Waltham, MA, USA), and proton magnetic resonance (‘Avance 400’ Bruker, Billerica, MA, USA).

*2,2,4-Trimethyl-1,3-dioxolane* (TMD): bp 98–99 °C, yield 83.7 mol % (isolated, on a theoretical basis). Found (%): C, 62.11; H, 10.48. Calculated for C_6_H_12_O_2_ (%): C, 62.04; H, 10.41. ^1^H–NMR (400 MHz, CDCl_3_): δ 1.18 (d, *J_HH_* = 6.0 Hz, 3H), 1.35 (s, 3H), 1.44 (s, 3H), 3.34-3.38 (m, 1H), 3.95–3.99 (m, 1H), 4.12–4.19 (m, 1H). ^13^C–NMR (100 MHz, CDCl_3_): δ 18.4, 26.1, 26.4, 70.8, 72.1, 108.6. The mass spectrum, *m*/*z* (*I*rel, %): 115 (47), 72 (23), 59 (18), 43 (100). GC: non-polar column, temperature ramp, Van den Dool and Kratz RI = 721, (poly(dimethylsiloxane), 30 m/0.32 mm/0.25 μm, He, 45 to 365 °C, 25 °C/min). UV-Vis spectroscopy: no band with ε more than 100 has been found in the spectrum range of 300–630 nm.

*2-Ethyl-2,4,5-trimethyl-1,3-dioxolane* (ETMD): bp 135–142 °C, yield 81.6 mol % (isolated, on a theoretical basis). Found (%): C, 66.69; H, 11.08. Calculated for C_8_H_16_O_2_ (%): C, 66.63; H, 11.18. The spectral data matched the literature data [66]. GC: non-polar column, the temperature ramp, Van den Dool and Kratz RI = 893, (poly(dimethylsiloxane), 30 m/0.32 mm/0.25 μm, He, 45 to 365 °C, 25 °C/min). UV-Vis spectroscopy: no band with ε more than 100 has been found in the spectrum range of 300–630 nm.

#### 3.2.2. The Synthesis of Glycol Ethers by Cyclic Ketal Reduction

The reduction of cyclic ketals with LAH-AlCl_3_ in ether was performed according to the protocol described by Legetter and Brown [29]. The products obtained after the ether distillation were double distilled before performing purity analysis.

*Isopropoxypropanols* (IPP, a mixture of 2-isopropoxy-1-propanol and 1-isopropoxy-2-propanol): bp 137–144 °C, yield 89.7 mol % (isolated, on a theoretical basis). Found (%): C, 60.87; H, 11.88. It has Calculated for C_6_H_14_O_2_ (%): C, 60.98; H, 11.94. ^1^H–NMR (400 MHz, CDCl_3_): δ 1.09–1.11 (m, 6H), 1.13–1.19 (m, 3H), 2.89–3.01 (m, 1H), 3.42–3.64 (m, 2H), 3.89–3.96 (m, 1H). ^13^C–NMR (100 MHz, CDCl_3_): δ 19.0 (17.7), 21.7 (22.2), 66.5 (66.0), 71.4 (70.6), 73.7 (72.5). The MS data matched the NIST main library spectra. GC: non-polar column, the temperature ramp, Van den Dool and Kratz RI = 800, (poly(dimethylsiloxane), 30 m/0.32 mm/0.25 μm, He, 45 to 365 °C, 25 °C/min). UV-Vis spectroscopy: no band with ε more than 100 has been found in the spectrum range of 300–630 nm.

*3-(sec-Butoxy)butan-2-ol* (SBB, a mixture of the diastereomers): bp 162–163 °C, yield 92.0 mol % (isolated, on a theoretical basis). Found (%): C, 65.88; H, 12.38. Calculated for C_8_H_18_O_2_ (%): C, 65.71; H, 12.41. ^1^H–NMR (400 MHz, CDCl_3_): δ 0.91 (t, *J_HH_* = 7.4 Hz, 3H), 1.08–1.13 (m, 6H), 1.16 (d, *J_HH_* = 6.4 Hz, 3H), 1.37–1.43 (m, 1H), 1.57–1.63 (m, 1H), 3.45–3.51 (m, 1H), 3.54–3.58 (m, 1H), 3.72–3.77 (m, 1H). ^13^C–NMR (100 MHz, CDCl_3_): δ 10.7, 16.5, 18.3, 19.4, 29.5, 70.2, 73.6, 76.6. Mass-spectrum, *m*/*z* (*I*_rel_, %): 101 (56), 73 (24), 57 (100), 55 (32), 45 (74). GC: non-polar column, temperature ramp, Van den Dool and Kratz RI = 984, (poly(dimethylsiloxane), 30 m/0.32 mm/0.25 μm, He, 45 to 365 °C, 25 °C/min). UV-Vis spectroscopy: no band with ε more than 100 has been found in the range of 300–630 nm.

### 3.3. Catalytic Experiments

The experiments of ketalization of the diols (1,2-PD and 2,3-BD) with ketones (acetone and MEK) under atmospheric pressure were carried out in a glass reactor (internal volume, 15 cm^3^) equipped with a thermostating jacket and a reflux condenser. A magnetic stir bar and the previously prepared reactant mixture (7 mL) were placed in the reactor and the thermostat was turned on. After reaching the set temperature, a catalyst charge was introduced into the reactor. This moment was taken as the initial reaction time. Stirring was periodically terminated to take aliquots of a 0.05–0.1 mL volume, which were analyzed by GLC. The following sampling protocol was applied: for each reaction equilibrium state 5–7 samples were taken; each sample was analyzed from 3 to 5 times to obtain the appropriate standard deviation (it did not exceed 0.2% for the diol conversion values from 65 to 95%).

The experiments on the cyclic ketals hydrogenolysis and the reductive alkylation were carried out in a stainless steel autoclave (internal volume of 50 mL, designed and manufactured by TIPS RAS, Moscow, Russia), equipped with a manometer (for pressure control) and a thermocouple. The specified amounts of the cyclic ketal (TMD or ETMD in the ketal hydrogenolysis) or the mixture of the diol and the ketone (acetone+1,2-PD or MEK+2,3-BD in the reductive alkylation), the catalyst sample, and a magnetic stir bar were placed in the autoclave. The aluminosilica-based Al-HMS catalysts were used in the oxide form after the calcination. The closed autoclave was purged with argon, then filled with hydrogen (to an initial pressure of 40 bar), and then placed in a furnace. The stirring was turned on, and this point of the time was taken as the onset of the reaction. At the end of the experiment time, the autoclave was cooled with the cold water, and the products of the catalysis were decanted, centrifuged and subjected to analysing by GC-FID (the pure samples used as the internal standards for quantification) and GC–MS. The catalyst was washed five times with acetone (the suspension was centrifuged and acetone was decanted each time), and then dried in a flow of argon. The heterogeneous catalyst, thus prepared, was used for the TEM study.

In the test of the catalyst reusability, after each cycle the catalyst was recovered in the same manner as for the characterization. The equal feed-to-catalyst weight ratio was maintained throughout the tests.

### 3.4. Catalysts

The commercially available sulfonic ion-exchange resin Amberlyst 36 dry (a sample has been kindly provided for testing by Dow Europe GmbH, Moscow, Russia) was used in the ketalization experiments without any prior modification. The preparation method details and the properties of Pd/Al-HMS catalyst have been reported elsewhere [39].

The textural characteristics of the synthesized aluminosilicates and the commercial supports were determined on the ASAP 2020 instrument from the company Micromeritics (Norcross, GA, USA) with the use of the low-temperature adsorption of nitrogen. The parameters were calculated by the BET method using the instrument software. The structure and morphology of the supported catalyst samples were studied by the high-resolution transmission electron microscopy (TEM) on a JEM 2100 electron microscope (from JEOL, Tokyo, Japan) at the accelerating voltage of 200 kV. The acid properties of the supports were studied using the temperature-programmed desorption of ammonia (TPD of NH_3_). The experiments were performed on an USGA-101 sorption analyzer from Unisit (Moscow, Russia). Before the adsorption of ammonia, the samples were in situ calcined in the flow of the dry air at 500 °C for 1 h and then cooled to 60 °C in the flow of nitrogen. The saturation was carried out in the flow of ammonia diluted with helium (1:1) at 60 °C for 30 min. The physically adsorbed ammonia was removed at 100 °C in the flow of helium for 1 h. The thermal desorption curves of ammonia were obtained at the temperature range from 100 °C to 800 °C in the flow of helium at the heating rate of 8 K/min.

### 3.5. Methods for Estimating Gasoline and Solvent Properties

#### 3.5.1. Physico-Chemical Properties

The main methods employed in the study on the solvent and gasoline blending properties have been summarized in Table 16.

The gross calorific values of the compounds were measured using a C200 calorimeter (IKA, Königswinter, Germany) according to the DIN 51900. The net calorific values were calculated regarding the hydrogen weight contents determined by the CHNS analysis.

In the water solubility measurements, water aliquots (0.01 g each) were added to a 3 mL aliquot of the organic compound with the periodical shaking until the phase separation had been visually observed. The similar procedure was used to measure the solubility of the organic compounds in water. To estimate polymer solubility, the specified polymer mass was brought in contact with the solvent (2.0 mL) with stirring at the temperature of 50–60 °C for 8 h.

An aliquot of the supernatant liquid was taken off and then evaporated in an argon flow (T = 150 °C, for 4 h) to give the residual mass of the polymer dissolved. Polystyrene (“402” trademark, Nizhnekamskneftekhim, Nizhnekamsk, Russia, *M*_w_ = 422280, *M*_n_ = 847060), polybutadiene (“CKDL” trademark, Nizhnekamskneftekhim, Nizhnekamsk, Russia, *M*_w_ = 680870, *M*_n_ = 1470180) and chlorinated poly(vinylchloride) (“PSH-LS” trademark chlorinated polyvinylchloride resin, Kaustik, Sterlitamak, Russia) were used as the polymer samples. logK_ow_ had been estimated using the CLogP group contribution method.

The saturated vapor pressure (SVP)—temperature dependences were obtained by the vacuum rectification of the compounds under the different residual pressures using the laboratory rectification column. The Antoine equation coefficients were calculated by means of the mathematical regression analysis.

The TGA evaporation rate measurements have been conducted using TGA/DSC3+ (Mettler Toledo, Columbus, OH, USA), with a TGA/HT DSC HSS2 detector. In a typical run the temperature was risen from 25 °C to 500 °C (the temperature ramp, 5 °C/min) under ambient air conditions (the flow of 20 mL min^−1^). The samples (20 μL volume) were put into the Pt crucibles (inner volume 70 μL) before the measurement. From the TGA curves, the temperatures corresponding to the 90% weight loss were determined. Butyl acetate (99.5%, Sigma Aldrich, St. Louis, MO, USA) was used as the reference substance.

#### 3.5.2. Solvatochromic Parameters Estimation

The parameter E_T_ was determined using 2,6-diphenyl-4-(2,4,6-triphenyl-1-pyridino)-phenolate (Reichardt betaine, 90%, Sigma-Aldrich, St. Louis, MO, USA) without the additional purification. 4-Nitroaniline (99%, Fluka, Buchs, Switzerland) and 4-nitroanisole (97%, Sigma Aldrich, St. Louis, MO, USA) were used to determine the parameters β and π*, respectively. All the ethers and ketals samples were stored over the 4A molecular sieves prior to the spectra acquisition in order to remove the trace water.

The concentration of the solvatochromic indicators was set at such the level that the absorption maximum was within the range of 0.2–1.0 absorbance units. The absorption spectra were recorded with the UV-3600 double-beam double-monochromator spectrophotometer (Shimadzu, Kyoto, Japan) equipped with a TCC-240A Peltier cell temperature control system. The temperature was maintained at a level of 25 ± 0.1 °C. The solutions were placed in quartz cells with optical path lengths of 10 or 1 mm (in the case of high background solvent absorption). The corresponding solvent without a solvatochromic indicator was used as the reference solution. The spectra were recorded within the range of 300–800 nm (depending on the indicator) at a spectral resolution of 1 nm. The distance between points in the spectrum was 0.1 nm. The obtained spectra were subjected to the Savitzky‒Golay smoothening with the subsequent localization of the long-wave absorption band λ_max_ (the precision was not worse than 0.1 nm). The Dimroth‒Reichardt energy (kcal/mol) was calculated using the following equation [67]:

(3) ET(30)=hcNAν˜maxB=28590λmaxB

where *h* is the Planck constant, *c* is the speed of light, *N*_A_ is the Avogadro number, ν_maxB_ and λ_maxB_ are the wavenumber (m^−1^) and wavelength (nm) at the Reichardt betaine absorption maximum, respectively. The normalized parameter *E*_T_ (30) in the solvent S was calculated by comparing the obtained value with the reference data for water and tetramethylsilane (TMS):

(4) $$E_{T}^{N}=\frac{E_{T}{\left(30\right)}_{S}-E_{T}{\left(30\right)}_{TMS}}{E_{T}{\left(30\right)}_{H_{2}O}-E_{T}{\left(30\right)}_{TMS}}=\frac{E_{T}{\left(30\right)}_{S}-30.7}{32.4}$$

The Kamlet‒Taft parameters π* and β were calculated using the literature data on the corresponding coefficients (b, s) for the location of the absorption bands of the studied solvatochromic indicators according to the equations [67]:

(5) $$\pi^{*}=0.427\left(34.12-{\tilde{\nu }}_{\max ANS}\right)$$

(6) $$\beta =\frac{31.10-3.14\pi^{*}-{\tilde{\nu }}_{\max ANI}}{2.79}$$

where ν_max ANI_ and ν_max ANS_ are the absorption maximum wavenumbers for 4-nitroaniline and 4-nitroanisole, respectively (in kK, 1 kK = 1000 cm^−1^). The parameter α was determined from the data on π* and the following equation:

(7) $$\alpha =0.186\left(10.91-{\tilde{\nu }}_{\max B}\right)-0.72\pi^{*}$$

## 4. Conclusions

Synthetic strategies for the cyclic ketal and the glycol ether derivatives of the renewable diols 1,2-propylene glycol and 2,3-butane diol have been proposed. The ketalization reaction has been subjected to a thermodynamic analysis. The homological reactions of the cyclic ketal formation from the diol and the ketone has been shown to comply with the isoequilibrium relationship. The ΔH^0^_r_/ΔS^0^_r_ (kJ mol^−1^; J mol^−1^ K^−1^) values for the reactions (1,2-propylene glycol + acetone → 2,2,4-trimethyl-1,3- dioxolane-+ water and 2,3-butanediol + 2-butanone → 2-ethyl-2,4,5-trimethyl-1,3-dioxolane) have been found to be −16.6±1.0/−56.8±3.3 and −15.1±0.9/−51.1±3.1, respectively.

The hydrogenolysis of the two cyclic ketals—TMD and ETMD—has been studied with the use of heterogeneous bifunctional catalysts obtained by doping Al-HMS mesoporous aluminosilicas with palladium metal. The catalysts have been successfully used for the synthesis of the glycol ethers via two different routes (the cyclic ketal hydrogenolysis and the reductive alkylation of the diol with the ketone): in both the cases the target products have been obtained with yields up to 91–93 mol %; the catalysts have been found to be reusable at least 5 times with a moderate decrease in activity (the yield of the target product on the 5th cycle had been registered as 84% of the first cycle yield).

The renewable diol derivatives (the two ketals—TMD and ETMD—and the two corresponding ethers—IPP and SBB) have been characterized by their solvent-relevant properties, including density, viscosity, boiling points, melting points, refraction coefficient and heat of combustion. The volatility properties have been investigated in order to obtain the relative evaporation rates (RER), as well as the Antoine equation coefficients.

TMD shows the possibility to evaporate considerably faster (RER = 1.30) than BuOAc (the reference substance), while ETMD, IPP and SBB may evaporate slightly slower (RERs 0.91, 0.84 and 0.73, respectively).

A solvent properties study has been conducted for TMD, ETMD, IPP and SBB, including their miscibility with organic solvents and water, their ability to dissolve polymers (polystyrene, polybutadiene and CPVC) and Kamlet-Taft solvatochromic parameters determinations. In the polymer dissolution tests, the cyclic ketals have been found to be remarkable solvents for polystyrene and polybutadiene: more than 300 g of the polymer per liter of the solvent could be dissolved. After positioning on β vs π* plots the conclusion has been made that the cyclic ketals (TMD and ETMD) appear to have close solvent properties to BuOAc, diethyl ether, eucalyptol and 1,1-diethoxy methane. The hydrolytic stability in the aqueous alkali media might be a possible advantage over BuOAc, while in the comparison with diethyl ether the ketals demonstrate considerably lower flammability hazards and potential evaporation losses for their lower volatility. Glycol ethers, IPP and SBB have similar solvent properties as C_5_-C_8_ fatty alcohols, what permits the alcohols (produced from ethylene via the Ziegler process) to be a potentially renewable alternative.

From the synthesis trees designed for the four compounds, the number of synthetic steps for TMD, ETMD, IPP and SBB has been found to be three, two, three and three, respectively. The primary sustainability estimate made for the synthetic protocols has shown positive results for most of the criteria. The problem to be solved here is the necessity of using palladium (an element at risk of the depletion) for the glycol ether synthesis by ketal hydrogenolysis.

We have also estimated the gasoline-blending properties of the renewable ketal glycol ethers. The blending research/motor octane numbers for TMD, ETMD, IPP and SBB have been found equal to 98.8/91.5, 93.8/91.5, 100.1/97.5 and 100.8/98.5, respectively. Thus, after subjecting ETMD to a mild and highly selective hydrogenolysis it has yielded an oxygenate with a substantially higher performance both in terms of its antiknock properties and hydrolytic stability.

SBB, with the highest boiling point (163–164 °C) among the oxygenates tested has not been found to affect gasoline volatility. The comparison between two derivatives of 2,3-BD (2-butanol and SBB) has shown that although the antiknock properties of the two compounds appear to be totally comparable, SBB has a particular advantage as far as the volumetric calorific value is concerned. After adding 10 vol. % of 2-butanol and SBB, the fuel AKI has increased from 88.2 to 89.2–89.3 points, while the NHOC for the blends have been 26.70 and 28.48 MJ·L^−1^ (6–7% more for the last mentioned one), respectively.

From the results obtained in the given investigation, a number of prospective future research directions may be outlined. The cyclic ketals and the corresponding glycol ethers (besides IPP being essentially an industrially available product) are reasonably estimated to be good thinners for dyes and the coating formulations. There is an example of the successful application of solketal (2,2-dimethyl-4-hydroxymethyl-1,3-dioxolane), a bio-based cyclic ketal being currently produced and utilized on the industrial scale.

The lower polarity and hydrophobicity of the diol ketals compared to solketal may help some of these compounds find applications in adjacent niches. For example, TMD and ETMD could be tested as possible replacements for conventional solvents such as THF and xylenes. One should mention SBB as the renewable isomer of petroleum-derived ethylene glycol monohexyl ether. Of course, the thorough toxicity and the environmental impact estimate are of particular interest.

Both the ketal hydrogenolysis and reductive alkylation approaches seem rather advantageous in terms of reaction selectivity and atom efficiency, except for the need of using a Pd-containing catalyst. It is thus of interest to substitute palladium with more sustainable alternatives, for example, nickel. These catalysts may be useful for homologous reactions, e.g., the reductive alkylation of furfural with lower alcohols.

## Figures and Tables

**Table 1:** The equilibrium compositions of the reaction mixtures obtained by the ketalization of 1,2-PD with acetone and 2,3-BD with MEK. The conditions: the ambient air pressure, keton:diol = 6:1 mol, 5 wt. % (to diol) of Amberlyst 36 dry as the catalyst.

*T*, K	*x* ^0^ *_ketone_*	*x* ^0^ *_diol_*	*x* ^1^ *_ketone_*	*x* ^1^ *_diol_*	*x* ^1^ *_ketal_*	*x* ^1^ *_W_*	*K_c_*	*X_eq_*
1,2-PD + acetone								
298	0.770	0.115	0.687	0.012	0.093	0.093	1.058	0.897
303	0.770	0.115	0.681	0.015	0.095	0.095	0.902	0.874
313	0.770	0.115	0.697	0.015	0.087	0.087	0.705	0.867
323	0.770	0.115	0.689	0.019	0.089	0.089	0.593	0.833
2,3-BD + MEK								
298	0.540	0.090	0.014	0.471	0.073	0.073	0.832	0.849
303	0.540	0.090	0.014	0.472	0.072	0.072	0.753	0.839
313	0.540	0.090	0.017	0.475	0.069	0.069	0.591	0.812
323	0.540	0.090	0.018	0.476	0.068	0.068	0.524	0.796
333	0.540	0.090	0.022	0.479	0.065	0.065	0.402	0.756

^1^ The *X*_eq_ data shown as the mean have been derived from the six samples ± SD (0.005 and 0.002 for 1,2-PD and 2,3-BD ketalization, respectively). *x*^0^_ketone_, *x*^0^_diol_ are the initial molar fractions of the ketal and the diol, respectively; *x*^1^_ketone_, *x*^1^_diol_, *x*^0^_ketal_, *x*^0^_W_ are the final (at the equilibrium state) molar fractions of the ketone, the diol, the ketal and water, respectively; *K*_c_ is the equilibrium constant; *X*_eq_ is the diol equilibrium conversion.

**Table 2:** The values of thermodynamic parameters for the diol ketalization reactions.

Reaction	Δ*H*^0^_r_, kJ mol^−1^	Δ*S*^0^_r_, J mol^−1^ K^−1^	Δ*G*^0^_r_, kJ mol^−1^	Reference
2,3-BD + MEK	−16.6 ± 1.0	−56.8 ± 3.3	0.30 ± 0.02	this study
2,3-BD + acetone ^1^	−16.7	−56.5	0.5	[47]
1,2-PD + acetone	−15.1 ± 0.9	−51.1 ± 3.1	0.05 ± 0.02	this study
1,2-PD + acetone ^1^	−3.3	−17.6	1.7	[47]
glycerol + acetone	−30.1 ± 1.6	−100.0 ± 10.0	−2.1 ± 0.1	[52]
glycerol + acetone	−14.5	−49.9	0.4	[54]
glycerol + acetone	−19.8 ± 1.6	−64.4 ± 5.2	−0.6 ± 0.05	[53]
glycerol + acetone ^1^	−15.5	−54.4	0.7	[47]
GMME ^2^ + acetone	−15.8 ± 1.2	−51.5 ± 3.9	−0.4 ± 0.03	[53]

^1^ The values for T = 300 K, the correlation coefficient *r* = 0.975–0.998; ^2^ No uncertainty data has been reported; ^3^ GMME = glycerol 1-monomethyl ether. The data has been shown as the mean ± SD.

**Table 3:** The physicochemical properties of the Al-HMS-supported catalysts.

Support	SiO_2_/Al_2_O_3_	S_BET_, m^2^ g^−1^	V_total_, cm^3^ g^−1^	Total Concentration of Acid Sites, mmol g^−1^	Pd Particles Mean Size, nm ^1^
Al-HMS(10)	9.8	680	0.7	256	3.8
Al-HMS(15)	14.7	770	0.9	211	4.2
Al-HMS(20)	19.7	850	1.0	170	3.5
Al-HMS(30)	29.6	820	1.1	144	4.1

^1^ The values for 2 wt.% Pd/Al-HMS supported catalysts have been calculated according to the TEM results; *S*_BET_ is the BET specific surface area; *V*_total_ is the total pore volume.

**Table 4:** The hydrogenolysis of TMD and ETMD over 2% wt. Pd/Al-HMS catalysts with the different supports. The conditions are: p(H_2_) = 40 bar, 100 mg of the catalyst, 2.5 mL of the ketal, the reaction time 5 h.

Entry No.	Support	T, °C	Y_IPP_, mol % ^1^	Y_SBB_, mol % ^1^
1	Al-HMS(10)	140	29.8	11.0
2		160	40.5	30.6
3	Al-HMS(15)	140	32.7	31.7
4		160	44.2	51.1
5 ^2^		160	91.4	92.1
6	Al-HMS(20)	140	38.6	33.3
7		160	53.2	35.6
8	Al-HMS(30)	140	19.9	31.3
9		160	13.9	25.3

^1^ Ketal to ether hydrogenolysis selectivity 97–98 mol % in all the cases; ^2^ reaction time, 24 h; *Y*_IPP_, *Y*_SBB_—the GC yields of IPP and SBB, respectively.

**Table 5:** The reductive alkylation of 1,2-PD and 2,3-BD with acetone and MEK with 2%Pd/Al-HMS(20) bifunctional catalyst. p(H_2_) = 40 bar, 10 mg catalyst, 2.5 mL of a diol.

Entry No.	Diol:Ketone Molar Ratio	T, °C	t, h	X_ketone_, %	Y_ketal_, %	Y_ether_, %	Y_alcohol_, %
MEK + 2,3-BD							
1	40	120	5	73.7	56.1	16.9	0.7
2	40	140	5	97.1	34.4	62.2	0.6
3	40	160	5	62.2	51.6	9.6	1.0
4	40	140	10	98.3	17.7	79.9	0.7
5	40	140	20	98.8	5.3	92.6	0.9
6	20	120	5	55.1	47.9	6.2	1.0
Ac + 1,2-PD							
7	40	120	5	86.0	70.2	15.2	0.5
8	40	140	5	96.3	36.5	58.7	1.1
9	40	160	5	82.4	71.1	7.9	3.4
10	40	140	10	97.8	15.4	81.3	1.1
11	40	140	20	98.0	3.6	93.3	1.1
12	20	120	5	85.0	77.2	6.8	1.0

*X*_ketone_—conversion of the ketone (MEK/acetone), *Y*_ketal_, *Y*_ether_, *Y*_alcohol_—the GC yields of the ketal (ETMD/TMD), the ether (SBB/IPP) and the alcohol (2-butanol/isopropanol), respectively.

**Table 6:** The synthesis of SBB over 2% wt. Pd/Al-HMS (15): the catalyst reusability test. The conditions are: p(H_2_) = 40 bar, 100 mg of the catalyst, 2.5 mL of the ketal, the reaction time was 5 h.

Cycle No.	*Y*_SBB_, mol %	
	ETMD Hydrogenolysis ^1^	2,3-BD Reductive Alkylation
1	35.6	62.2
2	36.9	54.7
3	35.3	53.3
4	35.1	53.1
5	32.5	52.1

^1^ Ketal to ether hydrogenolysis selectivity 97–98 mol % in all the cases; *Y*_IPP_, *Y*_SBB_—the GC yields of SBB.

**Table 7:** The main physicochemical properties of the ketals and the corresponding ethers.

Compound	bp, °C	mp, °C	d_20_, kg cm^−3^	n_D_^20^	*ν*_20_, mm^2^ s^−1^	*η*_20_, cP ^1^	NHOC, kJ/kg	NHOC, MJ/L
TMD	98–99	<−60	0.900	1.3940	0.75	0.68	28553 ± 43	25.70
ETMD	140–142	<−60	0.899	1.4110	0.23	0.20	31556 ± 12	28.37
IPP	144–145	<−60	0.883	1.4100	3.01	2.66	29839 ± 10	26.35
SBB	163–164	<−60	0.875	1.4175	3.89	3.40	32548 ± 5	28.48

^1^ The parameter was calculated from the experimentally measured density and the kinematic viscosity.

**Table 8:** The evaporation rates for the renewable diols derivatives calculated from the TGA measurements.

Compound	TMD	ETMD	IPP	SBB
*T*_10%_, °C	85.8	114.4	122.3	138.0
Evaporation rate, rel. to BuOAc	1.30	0.91	0.84	0.73

**Table 9:** The Antoine equation parameters for the 1,2-PD and 2,3-BD derived ketals and ethers.

Compound	Temperatures Range, K	A	B	C
TMD	298–334	3.9164	1280.14	−53.52
ETMD	318–372	4.1551	1618.36	−37.76
IPP	318–372	4.1812	1268.21	−108.16
SBB	324–386	5.2285	2039.98	−47.39

**Table 10:** The data on the water solubility of the renewable diol derivatives.

Equilibrium Solubility, wt. %	TMD	ETMD	IPP	SBB
Water in compound	1.8	0.3	∞	0.6
Compound in water	3.3	0.9	∞	3.8
Log*K_OW_*	1.46	2.51	0.40	1.24

**Table 11:** The IPP aqueous solutions densities and the melting points.

IPP Concentration, wt. %	d_20_, g/cm^3^	mp, ºC
100	0.883	<−60
80	0.925	−31.2
60	0.947	−22.5
40	0.959	−20.9
20	0.970	−14.7

**Table 12:** The equilibrium solubilities of the organic polymers in the 1,2-PD and 2,3-BD derivatives.

Polymer	Equilibrium Concentration (25 °C), g L^−1^			
	TMD	ETMD	IPP	SBB
Polystyrene	>300.0	>300.0	0.2	0.4
Polybutadiene	>300.0	>300.0	1.8	1.2
CPVC	8.9	11.3	1.9	1.7

**Table 13:** The Kamlet-Taft solubility parameters for the renewable diol derivatives.

Solvent	λ_max_, nm	$E_{T}^{N}$	λ_max_, nm	π*	λ_max_, nm	β	λ_max_, nm	α
IPP	572.2	0.595	304.8	0.56	367.6	0.77	572.2	0.81
SBB	626.2	0.462	303.3	0.49	364.9	0.77	626.2	0.58
TMD ^1^	-	-	300.5	0.36	349.8	0.50	-	-
ETMD ^1^	-	-	300.2	0.35	350.2	0.52	-	-

^1^ The data on *α* and $E_{T}^{N}$ is absent due to the abnormal behavior of the Reichardt’s dye in the ketal samples upon the spectra acquisition.

**Table 14:** The considerations of the renewable diols derivatives production sustainability.

Parameter	TMD	ETMD	IPP	SBB
Number of synthetic steps	3	2	3	3
Phosphorous compounds in the synthesis tree	none			
Highly hazardous compounds	none			
Volatile compounds of N, S, Cl, Br, F	none			
Very hydrophobic compounds with log*Kow* > 4.3	none			
Compound toxicity	no data			
Halogenated VOCs	none			
Elements at the risk of depletion in the synthesis	none		yes, palladium	
C_2_–C_7_ VOCs	yes, TMD	none	yes, IPP	none
Potentially renewable	yes			

**Table 15:** The gasoline blending properties of 1,2-PD and 2,3-BD ethers and ketals.

Property	Neat Gasoline	10% TMD	10% ETMD	10% IPP	10% SBB
*d*_20_, kg L^−1^	0.731	0.748	0.748	0.747	0.746
NHOC, MJ L^−1^	32.31	31.64	31.91	31.714	31.92
RON	91.8	92.5	92.0	92.7	92.7
bRON	-	98.8	93.8	100.8	100.8
MON	84.5	85.2	85.2	85.9	85.8
bMON	-	91.5	91.5	97.5	98.5
blending AKI ^1^		95.2	92.7	99.2	99.7
fractional composition, vol. %/°C					
ipb	35	36	40	36	35
5	45	49	50	46	44
10	52	57	57	53	50
20	61	68	73	68	63
30	72	80	86	80	80
40	85	91	103	94	94
50	104	107	121	114	114
60	124	120	135	127	135
70	142	136	146	139	149
80	160	157	160	150	161
90	186	192	187	175	182
fbp	193	194	190	190	189

^1^ The deriving made from the calculated bON values.

**Table 16:** The methods used for the diol derivatives characterization.

Property	Method	Instrument	Method Precision
Density at 20 °C	ASTM D4052	VIP-2MR vibration densitometer	±0.0001 g/cm^3^
Dynamic viscosity at 20 °C	ASTM D445	VPZh-2 glass viscosimeter	±0.0001 mm^2^*s^−1^
Refractive index at 20 °C n_D_^20^	-	IRF-22 refractometer	±0.0001
Melting point	DIN 51421	Kristall-20E	±1 °C
Saturated vapor pressure	ASTM D6378	Reid bomb, thermostate	±1.2 kPa
Fractional composition	ASTM D86	ARN-PKhP	±1 °C
RON/MON	ASTM D2700/ASTM D 2699	UIT-85	±0.1

## Supplementary Materials

The following are available online, Figure S1: Evaporation rates vs saturated vapor pressure (under 50 °C) plot, Figure S2: Evaporation rates vs boiling point plot; Table S1: Reduced pressure boiling points for TMD, ETMD, IPP and SBB; Table S2: Main physico-chemical properties of the base gasoline.

## Abbreviations

AKI: antiknock index; 2,3-BD, 2,3-butanediol; CPVC, chlorinated poly(vinyl)chloride; ETMD, 2-ethyl-2,4,5-trimethyl-1,3-dioxolane; 1,2-PD, 1,2-propanediol; IPP, isopropoxypropanol (an isomeric mixture of 2-isopropoxy-1-propanol and 1-isopropoxy-2-propanol); MEK, methyl ethyl ketone; MON, motor octane number; bMON, volumetric blending motor octane number; NHOC, net heat of combustion; RON, research octane number; bRON, volumetric blending research octane number; SBB, 3-(sec-butoxy)butan-2-ol (the mixture of diastereomers); SD, standard deviation; SVP, saturated vapor pressure; RER, relative (to *n*-butyl acetate) evaporation rate; TMD, 2,2,4-trimethyl-1,3-dioxolane.
