# Supplementary material for: Bio-Based Solvents and Gasoline Components from Renewable 2,3-Butanediol and 1,2-Propanediol: Synthesis and Characterization
Source: Molecules. 2020 Apr 9;25(7):1723. doi: 10.3390/molecules25071723 (PMC7180918; doi:10.3390/molecules25071723)
Supplement: Supplementary file 1 [file molecules-25-01723-s001.pdf]

molecules
MDPI

Table S1. Reduced pressure boiling points for TMD, ETMD, IPP and SBB.

**p, mmHg**	**bp, °C**
TMD	
37	25
67	39
74	41
88	45
110	50
131	54
148	57
167	61
ETMD	
20	45
31	55
38	68
58	75
79	83
97	87
124	90
145	92
165	99
IPP	
10	45
20	55
40	68
62	75
84	83
102	87
122	90
142	92
161	99
SBB	
5	51
11	63
22	76
37	87
57	96
80	103
93	107
120	113

Table S2. Main physico-chemical properties of the base gasoline.

Property	Value
Density ρ^{20}, g/cm^3	0.731
SVP, kPa	66.7
RON	91.8
MON	84.5
Fractional composition, vol. % distilled at temperature, °C:	
ibp	35
5	45
10	52
20	61
30	72
40	85
50	104
60	124
70	142
80	160
90	186
fbp	193
Hydrocarbon group content, vol. %	
isoalkanes	39.7
alkanes	16.6
arenes	39.7
cycloalkanes	3.8
alkenes	0.2
oxygenates	0.0
benzene	0.9

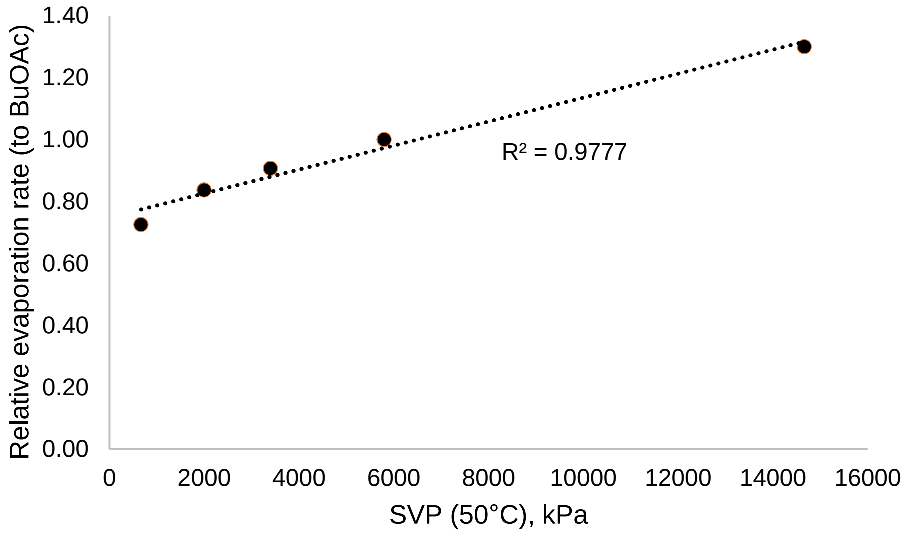

Figure S1. Evaporation rates vs saturated vapor pressure (under 50°C) plot. Bullets, left to the right: SBB, IPP, ETMD, BuOAc, TMD.

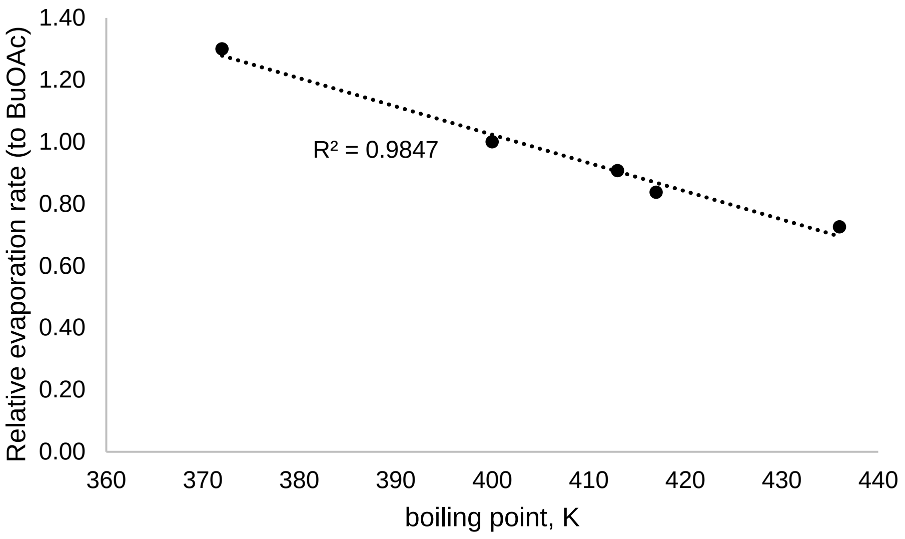

Figure S2. Evaporation rates vs boiling point plot. Bullets, from left to the right: TMD, BuOAc, ETMD, IPP, SBB.
